# Does the Severity of Arm Tremor in Essential Tremor Correlate with Impaired Balance?

**DOI:** 10.5334/tohm.1146

**Published:** 2026-02-03

**Authors:** Elan D. Louis, Diane Berry

**Affiliations:** 1 Department of Neurology, UT Southwestern Medical Center, 5323 Harry Hines Blvd, Dallas, TX 75390, USA; 2 Peter O’Donnell Jr. Brain Institute, University of Texas Southwestern Medical Center, Dallas, TX, USA

**Keywords:** essential tremor, clinical, tandem gait, balance, cerebellum

## Abstract

**Background:**

Motor features aside from tremor are increasingly recognized in essential tremor (ET) patients. The relationship between these features and tremor has received sparse attention. We examined whether the severity of action tremor in the arms was correlated with the severity of tandem gait difficulty and balance confidence.

**Methods:**

212 ET cases enrolled in a prospective clinical study, from which baseline data on the following variables were analyzed: severity of action tremor (total tremor score [TTS] from the Washington Heights-Inwood Genetic Study of Essential Tremor rating scale, range = 0–36 [severe tremor]), tandem gait mis-steps (range = 0–10), and Activities of Balance Confidence (ABC-6) Scale (range = 0 [least confident] – 100).

**Results:**

Higher TTS was associated with a greater number of tandem gait mis-steps (Spearman’s rho = 0.216, p = 0.002) and higher tertile of number of tandem gait mis-steps (Spearman’s rho = 0.237, p < 0.001). Higher TTS was associated with reduced balance confidence (i.e., lower ABC-6 score) (Spearman’s rho = –0.196, p = 0.004) and lower tertile of balance confidence (Spearman’s rho = –0.175, p = 0.01).

**Discussion:**

We report an association between the severity of upper limb action tremor in ET and both a self-reported measure of balance confidence and a performance-based measure of balance. These data support the model that upper limb action tremor and tandem gait difficulty are associated in some way, with one possible interpretation being that they are both related to a common underlying element, cerebellar dysfunction.

## Introduction

Action tremor of the arms is the primary clinical feature of essential tremor (ET) [1,2,3,4]. In some classification schemes [5], this tremor is cast as the sole clinical feature of ET. Within this framework, one could regard action tremor in ET as an isolated, self-contained entity. Indeed, an older literature cast ET as a monosymptomatic disease [6]. However, studies over the past two decades increasingly document a range of additional motor features in ET patients [6]. Despite this, analyses of the relationship between these features and the cardinal motor feature of the disease, action tremor of the arms, are sparse [7,8,9,10,11], and most of these analyses do not adjust for confounding factors [9,10,11]. Hence, there is an identifiable gap in knowledge.

This knowledge is important, as it informs our ability to address certain biological questions about ET. For example, if action tremor of the arms in ET were correlated with another motor feature, it would suggest a common pathophysiological basis. By analogy, in Parkinson’s disease (PD), the severity and therapeutic responsiveness of bradykinesia and rigidity are generally correlated with one another, whereas the severity and therapeutic responsiveness of rest tremor and gait and balance difficulty seem to be unrelated to those of bradykinesia and rigidity. That lack of correlation has led investigators to posit that these clinical features arise from different underlying biological processes and/or neurotransmitter systems [12,13,14,15]. In PD or ET, knowing whether or not treatable features are biologically linked, organizes and guides ones approach in the search for novel pharmacotherapies [16].

An emerging motor feature of ET is gait ataxia [17], which is generally mild, but can approach moderate levels of severity [18]. This gait ataxia is not benign – it is associated with lower balance confidence and more near falls [4,19] as well as increased risk of mortality [20]. A reasonable physiological construct is that both this ataxia and the action tremor of the arms in ET are grounded in cerebellar dysfunction [4,19]. If this model were correct, one would hypothesize that the two would track with one another – if one is severe in patients, the other will be as well. To our knowledge, there is no published study with a central focus on this question. There are a handful of studies, however, that have focused on other questions, but which have presented relevant data [7,8,9,10,11]. Most of these did not did adjust for confounding factors [9,10,11]. Furthermore, the results of these studies, in aggregate, are mixed [7,8,9,10,11]. Here, we leverage data from a clinical study involving more than 200 ET cases, addressing the specific question: is the severity of action tremor of the arms in ET correlated with the severity of tandem gait difficulty and frequency of self-reported falls? These data are expected to improve our understanding of underlying disease processes.

## Methods

### Study Overview

Our initial sample comprised 380 ET cases enrolled in a prospective, longitudinal study of cognition in elders with ET (COGNET: Clinical Pathological Study of Cognitive Impairment in Essential Tremor, National Institutes of Health Award #R01-NS086736) [21]. Nationwide enrollment commenced in July 2014. To date, cases reside in 43 US states. Eligibility requirements were as follows: (1) a diagnosis of ET, (2) a baseline age ≥ 55 years, and (3) enrollment as an eventual donor in the Essential Tremor Centralized Brain Repository. The Yale University, Columbia University, and University of Texas Southwestern Medical Center Institutional Review Boards approved the study protocol, approval number STU2020–0564, and all cases provided written, signed, informed consent.

Evaluations took place at baseline (T1) and then at 18-month intervals (T2 through T6), with the cohort followed for 10 years. The current analyses report baseline data, as this was the evaluation with the largest sample size.

A trained research assistant administered evaluations during home visits, except during the 2020–2021 COVID pandemic, when some evaluations were conducted remotely via Zoom. Each visit involved the completion of demographic and clinical questionnaires, participation in a videotaped neurological examination, and completion of a battery of neuropsychological tests. Evaluations, which typically lasted 4–5 hours, were usually completed in two sessions on one day (i.e., morning, afternoon) or on two adjacent days.

### Demographic and Clinical Questionnaires

Cases provided demographic information (age, sex, education) as well as a tremor history (e.g., age of tremor onset, tremor duration, use of ET medications). The six-item Activities of Balance Confidence (ABC-6) Scale was administered, which asked cases to rate their confidence in performing six tasks (e.g., reaching on tiptoes for an object) without losing balance or becoming unsteady [22]. Scores for each individual item ranged from 0 (least confident) – 100, with the total score (0–100) being the mean of the six item scores.

### Neurological Examination and ET Diagnoses

A 20-minute videotaped neurological examination was performed at each evaluation [23]. The severity of postural tremor (one examination item) and kinetic tremor (five examination items [drawing spirals, finger-nose-finger maneuver, pouring, drinking, using a spoon]) was assessed in each arm. An experienced movement disorders neurologist (E.D.L.) used the Washington Heights-Inwood Genetic Study of Essential Tremor (WHIGET) rating scale to rate these tremors (0 [none], 0.5 [minimal], 1 [mild], 1.5 [mild to moderate], 2 [moderate], 3 [severe]), resulting in a total tremor score (range = 0 [low severity] to 36 [high severity]) [24], which is both a reliable [25] and valid rating scale [26]. Detailed analyses have revealed substantial strength of association (*p’s* ≤ 0.001, mean *r* = 0.89) and substantial to near perfect agreement (mean *k* = 0.86, range = 0.64 to 1.00) between items rated with the WHIGET and Tremor Research Group Essential Tremor Assessment Scale (TETRAS) [27], indicated that ratings provided by each scale are highly comparable [28].

Based on a review of the questionnaire and videotaped examination data, the neurologist confirmed clinical diagnoses of ET using WHIGET criteria [29]. At a minimum, possible ET required moderate or greater amplitude kinetic tremor on examination during at least three activities, in the absence of another known cause (e.g., medication-induced tremor and tremor from hyperthyroidism). PD was diagnosed using the published diagnostic criteria, which required the presence of at least two cardinal signs [30,31].

### Tandem Gait Assessment

To assess tandem gait, cases were asked to walk 10 steps on a straight path with the heel of the leading foot touching the toe of the following foot. The number of mis-steps, defined as steps off the straight line, was the reported outcome [32]. If dependence on a cane, walker or wheelchair prevented the participant from completing one step safely, their performance was coded as 10 mis-steps.

### Cognitive Assessment

Based on a detailed neuropsychological assessment, all cases were assigned a diagnosis of either normal cognition, mild cognitive impairment, or dementia, as described previously [33,34].

### Final Study Sample

There were 380 participants. We excluded participants for the following reasons: diagnosis of ET was not confirmed (n = 36), diagnosis of PD (n = 3), diagnosis of dementia (n = 23), incomplete data on tandem mis-steps (n = 76, the vast majority of these took place during the 2020–2021 COVID pandemic, when some evaluations were conducted remotely, and gait testing could not be safely conducted), or incomplete data on one or more confounding variables (n = 30). The final sample comprised 212 ET cases. These 212 cases were compared to the 168 excluded participants; they were similar in age (median = 80.0 years for each, *p* = 0.16), sex (61.3% vs. 57.7% women, *p* = 0.48), educational attainment (median 16.0 years for each, *p* = 0.82), proportion ever prescribed a tremor medication (85.4% vs. 80.4%, *p* = 0.19), total tremor score (median = 23.5 vs. 22.9, *p* = 0.10), and number of tandem mis-steps (median = 4.0 for each, *p* = 0.09).

### Statistical Analysis

All analyses were performed in SPSS (Version 30.0). Descriptive statistics were calculated, with medians and interquartile ranges presented when the variable was not normally distributed (Table 1). Spearman’s correlation coefficients assessed the correlation between total tremor score and number of tandem mis-steps as well as the correlation between total tremor score and ABC-6 score; these analyses included 95% confidence intervals in some instances. Total tremor score was subdivided into kinetic tremor score (range = 0–30) and postural tremor score (range = 0–6). Partial Spearman’s correlations were conducted controlling for the potential confounds of sex and number of prescription medications reported. When considering age as a confounder, we age-restricted our sample to 170 ET cases with a narrower age range (65–90 years) to reduce the effects of high collinearity between age and primary variables of interest. In several analyses, we created tertiles of the variable of interest (e.g., tandem mis-steps, ABC-6 score, total tremor score) to gauge the behavior of correlations of interest in the setting of step-wise increases in these variables of interest.

**Table 1:** Demographic and clinical features of 212 ET cases.


FEATURE	DATA

Age (years)	80 [12], range = 55–95

Female Sex	130 (61.3)

Education (years)	16 [4]

Total number of current prescription medications	5 [4]

Family history of ET	108 (50.9)

Ever prescribed ET medications	181 (85.4)

Age of tremor onset (years)	41 [42]

Tremor duration (years)	34 [37]

Total tremor score	23.5 [7.5], range = 8.5–36

Number of tandem mis-steps	4.0 [9], range = 0–60

ABC-6 score	61.6 [45.0], range = 0–100


Values are median [interquartile range] or number (percentage) unless otherwise specified.

ABC = Activities Balance Confidence Scale, ET = essential tremor.

## Results

### Case Characteristics

The 212 ET cases had a median age of 80 years (Table 1). The median total tremor score = 23.5 (interquartile range = 7.5, range = 8.5–36), indicating moderate to severe tremor on average, within a wide range. The median number of tandem mis-steps = 4 (interquartile range = 9, range = 0–10) and the median ABC-6 score = 61.6 (interquartile range = 45.0, range = 0–100), indicating a wide spectrum of balance capabilities (Table 1).

### Association Between Tremor Severity and Number of Tandem Mis-steps

**Figure 1:**
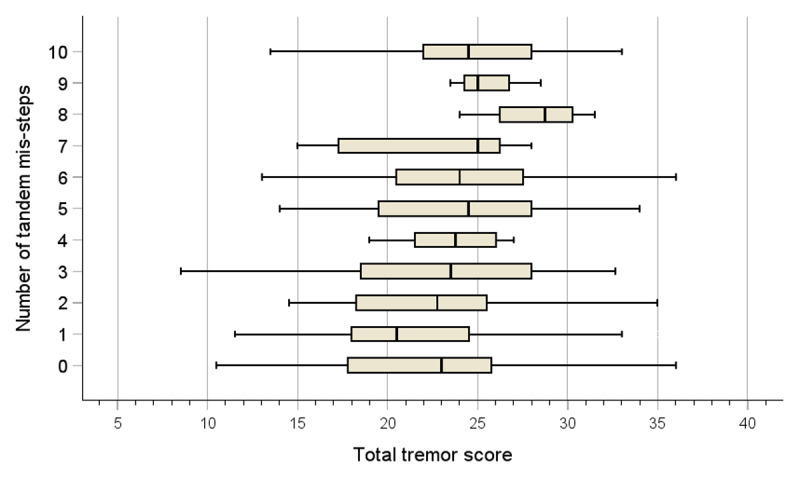
Total tremor score by number of tandem mis-steps in 212 ET cases. Each horizontal bar represents the median value, interquartile range and range at each level of tandem mis-steps.

Greater tremor severity (i.e., total tremor score) was associated with a greater number of tandem mis-steps (Spearman’s rho = 0.216, *p* = 0.002). Figure 1 shows a subtle and gradual increase in total tremor score (i.e., increase in tremor severity) with increasing number of tandem mis-steps.

Number of tandem mis-steps was similarly associated with kinetic tremor severity (Spearman’s rho = 0.231, 95% confidence interval = 0.110–0.345, p < 0.001) and postural tremor severity (Spearman’s rho = 0.257, 95% confidence interval = 0.138–0.368, p < 0.001).

**Figure 2:**
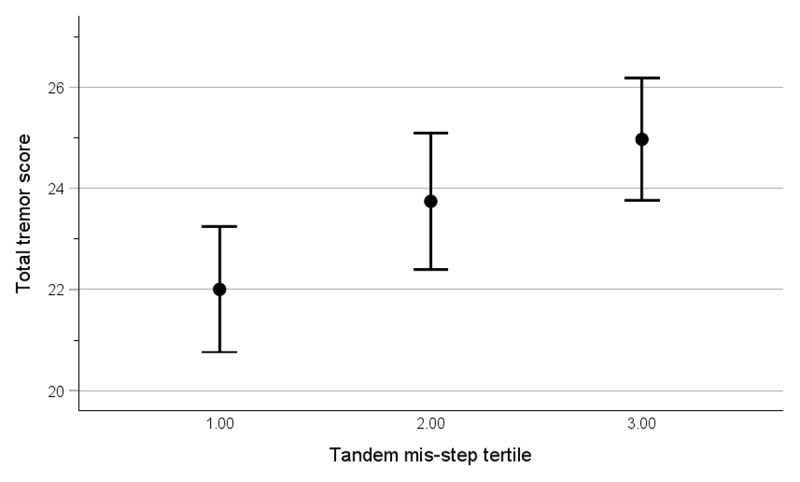
Total tremor score by tandem mis-step tertile in 212 ET cases. Vertical bars present mean ± 2 standard errors.

We created tertiles of tandem mis-steps to gauge the behavior of correlations of interest in the setting of step-wise increases in this variable. Tertiles of tandem gait difficulty were as follows: 0–2 tandem mis-steps (n = 82), 3–7 tandem mis-steps (n = 69), and 8–10 tandem mis-steps (n = 61). The mean ± standard deviation total tremor score in each tandem mis-step tertile was: 22.0 ± 5.6 [median = 22.5] in the lowest tertile, 23.7 ± 5.6 [median = 24.5] in the intermediate tertile, and 25.0 ± 4.7 [median = 24.5] in the highest tertile (Spearman’s rho = 0.237, *p* < 0.001). Figure 2 portrays the increase in total tremor score with each tandem gait mis-step tertile. We also created tertiles of total tremor score (≤ 21 [n = 73], > 21–25.5 [n = 66], and > 25.5 [n = 73]). The mean ± standard deviation number of tandem mis-steps in each total tremor score tertile was: 3.4 ± 3.5 [median = 2] in the lowest tertile, 5.0 ± 4.0 [median = 4] in the intermediate tertile, and 5.4 ± 3.8 [median = 5] in the highest tertile (Spearman’s rho = 0.214, *p* = 0.002).

A number of confounders were considered because they have been shown to be associated with either total tremor score, tandem gait difficulty, or both [8,9]. These are age, sex, and total number of current prescription medications. The association between total tremor score and number of tandem mis-steps remained significant after adjusting for sex and total number of prescription medications (Partial correlation coefficient, Spearman’s rho = 0.187, *p* = 0.007). Because of the collinearity between age and tandem mis-steps in this sample (Spearman’s rho = 0.50, *p* < 0.001), for this analysis, we age-restricted our sample to 170 ET cases between ages 65 and 90 years; in that analysis, the association between total tremor score and number of tandem mis-steps remained significant after adjusting for age, sex and total number of current prescription medications (Partial correlation coefficient, Spearman’s rho = 0.175, *p* = 0.02).

### Association Between Tremor Severity and Balance Confidence

Greater tremor severity (i.e., total tremor score) was associated with reduced balance confidence (i.e., ABC-6 score) (Spearman’s rho = –0.196, *p* = 0.004).

Reduced balance confidence was similarly associated with kinetic tremor severity (Spearman’s rho = –0.175, 95% confidence interval = –0.282 – –0.064, p < 0.001) and postural tremor severity (Spearman’s rho = –0.243, 95% confidence interval = –0.343 – –0.137, p < 0.001).

**Figure 3:**
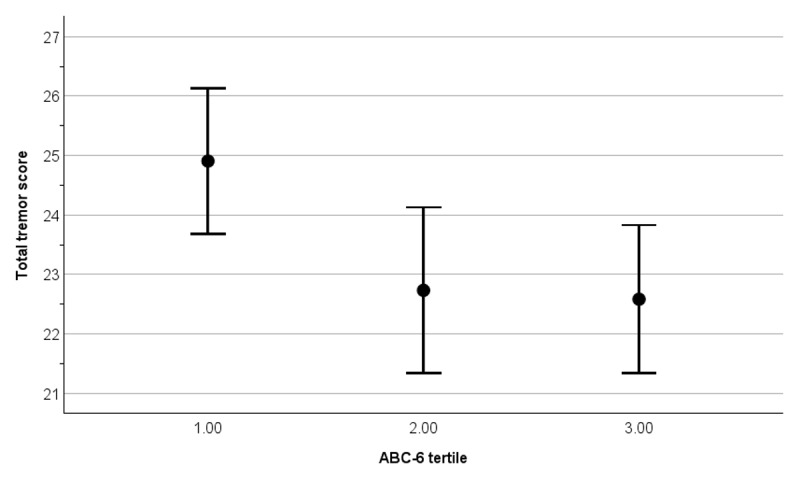
Total tremor score by ABC-6 tertile in 212 ET cases. Vertical bars present mean ± 2 standard errors.

We created tertiles of ABC-6 score (≤ 45 [n = 72], > 45–74 [n = 68], and > 74 [n = 72]). The mean ± standard deviation total tremor score in each ABC-6 score tertile was: 24.9 ± 5.2 [median = 25] in the lowest balance confidence tertile, 22.7 ± 5.8 [median = 23] in the intermediate tertile, and 22.6 ± 5.3 [median = 23] in the highest balance confidence tertile (Spearman’s rho = –0.175, *p* = 0.01), indicating that lower balance confidence tertile was associated with higher total tremor score. Figure 3 portrays the inverse correlation between total tremor score and ABC-6 score (i.e., greater tremor was associated with lesser balance confidence).

We also created tertiles of total tremor score (≤ 21 [n = 73], > 21–25.5 [n = 66], and > 25.5 [n = 73]). The mean ± standard deviation balance confidence in each total tremor score tertile was: 64.5 ± 23.9 [median = 68.3] in the lowest total tremor score tertile, 53.0 ± 28.0 [median = 53.3] in the intermediate tertile, and 52.8 ± 28.0 [median = 56.7] in the highest total tremor score tertile (Spearman’s rho = –0.173, *p* = 0.01).

The same set of confounders was considered as was considered for number of tandem mis-steps. The association between total tremor score and ABC-6 score remained significant after adjusting for sex and total number of current prescription medications (Partial correlation coefficient, Spearman’s rho = –0.172, *p* = 0.013). Because of the collinearity between age and ABC-6 score in this sample, we age-restricted our sample to 170 ET cases between ages 65 and 90 years; in that analysis, the association between total tremor score and ABC-6 score remained significant after adjusting for age, sex and total number of current prescription medications (Partial correlation coefficient, Spearman’s rho = –0.156, *p* = 0.04).

## Discussion

We report an association between the severity of upper limb action tremor in ET and both a self-reported measure of balance confidence as well as a performance-based measure of balance. Although the correlation coefficients were statistically significant, their magnitude was modest. This is not surprising. As discussed below, the association between upper limb action tremor and impaired balance in ET is likely not a direct causal one characterized by a large correlation coefficient. Rather, upper limb action tremor and impaired balance in ET are each likely the result of a third (i.e., common) underlying element – cerebellar dysfunction.

The existing literature on this topic is surprisingly sparse [7,8,9,10,11]. It is noteworthy that none of the handful of published papers on this topic was designed with the focus of assessing the association between tremor severity and tandem gait difficulty. Nonetheless, embedded in each of these papers are sub-analyses with relevant data that can be used to address this question. Thus, in a study 358 ET patients in China, arm action tremor score was correlated with number of tandem mis-steps (rho = 0.188, p < 0.001); however, in the final adjusted regression model, arm action tremor score was not associated with tandem gait abnormality (defined as ≥ 2 tandem mis-steps) [7]. In a smaller study of 30 ET patients in the Czech Republic, among whom upper limb accelerometry was performed, the mean acceleration amplitude of kinetic tremor was 2.48 cm/s^2^ in patients with no tandem gait mis-steps, 3.11 cm/s^2^ in patients with 1 mis-step, and 4.16 cm/s^2^ in patients with ≥ 2 mis-steps; however, this numerical difference was neither subjected to statistical testing nor were analyses adjusted for confounding effects (e.g., age) [10]. In a study of 122 ET patients in the US, total tremor score was not correlated with the number of tandem mis-steps (rho = 0.01, p = 0.89). The mean age of those patients was 64.9 years and mean total tremor score was 18.1 – a younger sample with less severe tremor than the patients in our current study [11]. Adjusted analyses were not performed. In another study of 120 ET patients in the US, total tremor score was not correlated with the number of tandem mis-steps (rho = 0.14, p = 0.15) in an unadjusted analysis [9]. The mean age of these cases was 71.3 years and mean total tremor score was 20.3 [9], also indicating a younger sample with less severe tremor than in the current study. Adjusted analyses were not performed. In an earlier study of the current cohort, conducted in a smaller subsample of 149 cases with both baseline and follow-up data, we assessed the baseline predictors of “more tandem mis-steps” at the second follow-up assessment [8]. “More tandem mis-steps” was defined based on the median number of tandem mis-steps [8]. In an unadjusted logistic regression model, total tremor score was associated with more tandem mis-steps (OR = 1.12, 95% confidence interval = 1.04–1.21); however, in a logistic regression model that adjusted for age and medications, OR = 1.08, 95% confidence interval = 0.99–1.18, indicating a marginal assocation [8].

In summary, four [7,8,9,11] of five prior studies reviewed above performed statistical testing and only two [7,8] of these performed adjusted models. Among the four prior studies that performed statistical testing [7,8,9,11], there was an association between tremor severity and tandem mis-steps in two [7,8]. Among the two that performed adjusted analyses [7,8], neither reported an association between tremor severity and tandem mis-steps, although the results were marginal in one [8]. These data present a picture of a small number of studies, most of which did not present adjusted analyses, with a mixed set of results, and no reported association in adjusted analysis. The added value of our current analyses is that they focused on the specific question at hand, relying on a large sample of more than 200 ET cases, and the analyses assessed several measures of balance and incorporated adjusted models. The data show a correlation that is modest in magnitude (e.g., for tandem mis-steps, the partial correlation coefficient, Spearman’s rho = 0.175) but significant (p = 0.02).

These data provide evidence that upper limb action tremor and tandem gait difficulty are associated in some way, with one possible interpretation being that they are both related to the common underlying element of cerebellar dysfunction. Other data to support a common basis of these two features. For example, tandem gait difficulty in ET improves with low doses of ethanol [35], as does the arm tremor in ET [36], again suggesting that these two clinical features share a common mechanistic (i.e., pharmacological) basis.

In these analyses, we demonstrate an association between the extent of upper limb action tremor and the extent of gait impairment in ET. Interestingly, though, thalamic stimulation can lessen tremor severity yet at the same time, it can worsen gait ataxia in ET. It is important to note that thalamic stimulation is a *therapeutic intervention* rather than a disease. ET is a *disease*. The two are not equivalent constructs. In the disease (i.e., ET), as demonstrated here, upper limb action tremor severity is correlated with gait ataxia because presumably both are due to cerebellar degeneration. In the setting of the intervention; however, there is a dissociation – the intervention can improve tremor severity yet worsen gait ataxia. Hence, the intervention has different impact on these two symptoms of cerebellar degeneration in ET. Understanding why this is the case would require greater understanding of the mechanism of action of this therapy.

This study was not without limitations. First, 76 ET cases were excluded due to incomplete data on tandem mis-steps. The vast majority of these were evaluated during the 2020–2021 COVID pandemic, when some visits were conducted remotely, and gait testing could not be safely conducted. We demonstrated that our final sample of cases did not differ from those excluded participants with respect to any demographic or clinical characteristics, indicating that our final group was representative of the larger pool from which they were drawn. Second, we did not perform accelerometry or quantitative gait testing, which would have provided more objective and precise measures of tremor severity and tandem gait difficulties. This relative lack of precision likely biased our results *towards* the null hypothesis, making it *more difficult* for us to detect an association. Therefore, it is possible that the associations we report are greater than we have estimated.

The study had a number of strengths. This is the only study to date to have focused on this particular association. Second, we enrolled a large sample of more than 200 ET cases, providing ample power to detect subtle associations. Third, our analyses included both a measure of self-reported balance difficulty and an objective measure of gait as well. Fourth, we carefully adjusted for the potential effects of relevant confounding factors.

In summary, we report an association between the severity of upper limb action tremor in ET and both a self-reported measure of balance confidence as well as a performance-based measure of balance. These data lend support to the physiological construct that in ET, the action tremor of the upper limbs and ataxia are both grounded in the same underlying dysfunction. As such, these data provide additional insights into our understanding of underlying disease processes. They also open the door to the notion that future novel treatments for the upper limb tremor in ET might be evaluated as potential treatments for the gait ataxia well.
